# Marketization in Crisis: The Political Economy of COVID-19 and the Unmaking of Public Transport in Stockholm

**DOI:** 10.1177/08969205211069862

**Published:** 2023-03

**Authors:** Alexander Paulsson, Till Koglin

**Affiliations:** Lund University, Sweden

**Keywords:** public transport, marketization, neoliberalism, Sweden, COVID-19

## Abstract

While measures to prevent the spread of COVID-19 disturbed both global and local markets, some commentators also argued that the pandemic could be seen as the beginning of the end of neoliberalism. Although neoliberal reforms have come under pressure, little is known about the implications of COVID-19 in or across specific sectors. Scaling down the rich theoretical–historical debates about neoliberalism to the regional level, we study the impact of COVID-19 on the marketized public transport system in Stockholm, Sweden. During COVID-19, ridership dropped as did ticket revenues, which put the market under operational and financial distress. Drawing on a discussion of the *norms* and *techniques* of marketization, we probe how the contracted bus operators responded to the pandemic, how they tried to save the market from collapsing, and whether the measures taken suggest an organized move away from neoliberal policies. Adding to recent debates of COVID-19 and neoliberalism’s longevity, we conclude that although the *norms* underpinning marketization remained unquestioned, the *techniques* were partly re-evaluated in the midst of the global crisis as a way to protect the established neoliberal policies from falling apart.

## Introduction

Due to COVID-19, the number of passengers using public transport in Stockholm decreased dramatically. During March 2020, the metro lost 20% of its passengers and for bus operations, it was a corresponding figure. On 19 March, the regional Public Transport Administration (PTA) in Stockholm issued a press release, stating that the supply of bus traffic would be cut. Due to bus drivers and other staff being on sick leave, the regional PTA, in consultation with the bus companies, decided to reduce the number of departures starting from 23 March. Fredrik Cavalli-Björkman, the director of the PTA, explained in a press release that:

I believe that our travelers will show understanding for the measures we are now forced to implement, they have shown this by taking seriously the authorities’ advice and taking responsibility for reducing the spread of the new coronavirus. [. . .] As fewer people than usual travel by public transport, we expect that there will be no increased congestion despite less frequent traffic. (Trafikförvaltningen, 2020a)

Kristofer Tamsons, a leading politician representing the conservative party (Moderaterna) in the transport council, and thereby responsible for public transport in the metropolitan region of Stockholm, re-published this message on his personal Facebook page. But the decision to cut down on traffic was soon criticized. The unions organizing the bus drivers argued that their members wanted to continue to work, but could not do so due to canceled bus departures (Rogvall, 2020a). Only days after cutting down bus traffic, on-board congestion increased (Rogvall, 2020b; Rogvall et al., 2020). Asked by a journalist about the cut in bus operations, Anders Tegnell, the state epidemiologist, who was seen daily in the media at the time, replied that it was ‘unnecessary to reduce that activity now’ (Holm, 2020). This had an effect. The next day, Tamsons (2020) wrote on Facebook:

I do not want to hear about any summer schedules or restless bus drivers. Drive everything you can, as much as you can. Bring in temporary employees, demand overtime and call-in other drivers who can line up. This is not a recommendation. It’s an order. This is the overall policy.

This message was also reproduced in other media [see e.g. Tihinen, 2020]. Bus operations commenced again with more frequent departures, but not everywhere and not according to the original timetable. Difficulties in updating the SL app [PTA’s combined travel and ticket app], and thereby communicating the new departure times, meant that many travelers still had to ride on crowded buses during peak hours. At the same time, there were fewer travelers across the entire system. And with fewer travelers, ticket revenues decreased as did the rate of profit.

Before the COVID-19 pandemic, public transport in Stockholm had been undergoing intense, yet slow, transformations of marketization for many decades. Since the early 1990s, public transport has moved from being organized as a municipal monopoly to a regionalized market, with multinational corporations competing for contracts, where the winning bidder then gets to operate the traffic on behalf of the PTA on 10-year contracts (Paulsson, 2020). One key policy innovation in this transformation was the development and application of so-called incentivized contracts, in which bus companies receive financial enumeration based on the number of boarding passengers. COVID-19 led to heated discussions within the public transport industry, especially on the question of how the incentivized contracts should be interpreted and applied. As this *technique* of public transport marketization experienced an unprecedented crisis, this raised another set of questions: Was the far-flung *norms* of marketization criticized and if so, by whom and in what respects (e.g. Araujo, 2007; Kjellberg and Helgesson, 2007)?

COVID-19 has also triggered a debate about the longevity of neoliberalism and whether the pandemic could be seen as the beginning of the end of this political regime of economic accumulation (Harvey, 2020; Kılıç, 2020; Navarro, 2020). Several commentators have earlier also pointed to the unexpected longevity of neoliberalism, most recently Plehwe et al. (2020) in their edited volume, *Nine Lives of Neoliberalism*. After the 2008 financial crisis, neoliberalism was deemed ‘undead’ as it had this strange ability to return from the grave (e.g. Crouch, 2011). Descriptions such as ‘zombie capitalism’ also circulated at the time (Harman, 2010). While COVID-19 has had devastating consequences for people’s health, it has also reawakened the debate about neoliberalism’s longevity and whether the pandemic is the potential beginning of the end of neoliberalism (e.g. Harvey, 2020; Kılıç, 2020; Nunes, 2020). Although much of this debate has dealt with the non-resilience of healthcare systems under neoliberal policies (e.g. Jagannathan and Rai, 2021), COVID-19 also had a major impact on public transport markets.

Drawing upon theories of marketization as a form of neoliberal transformation, we set out to explore how the crisis disclosed the underlying *techniques* and *norms* of marketization of public transport in Stockholm, Sweden.^1^ By exploring these questions, we will also be able to probe how neoliberalism, at least at the subnational level, manages to reinvent itself, save itself from collapsing, and possibly coming out stronger at the end of the crisis.

With more than 5-years’ experience of doing research on public transport markets, we conducted interviews in the autumn of 2020 with CEOs and senior managers of bus companies and civil servants in senior positions, working for the public transport authorities in Sweden. A total of 10 interviews were conducted and the interviews averaged 35 minutes (see ‘List of interviewees’ in Appendix 1 for further information). Due to the pandemic, all interviews were conducted either via Zoom or Teams. Many interviews were recorded, but not all. Of those who were not recorded, notes were taken. All interviews followed a semi-structured template.

## COVID-19, neoliberalism, and marketization

The COVID-19 pandemic has fundamentally affected both society and public transport. In Sweden, public transport is organized as a public market, where price competition takes place through secret tenders in the procurement process. While EU directives and national legislation shape the procurement process, another policy has emerged from the market participants themselves: incentivized contracts. These contracts are supposed to incentivize the bus companies to increase the number of passengers and act more business-like. Linking performance to financial enumeration is thought to improve quality and increase the bus companies’ revenues.

Scaling down the rich theoretical–historical debates about neoliberalism as a hegemonic world system (Slobodian, 2018; Springer et al., 2016) to the subnational and regional levels, the norms and techniques of marketization come to the foreground. These norms and techniques are key to understanding how neoliberal policies operate. This is also echoed in Harvey’s (2005) definition of neoliberalism:

Neoliberalism is in the first instance a theory of *political economic practices* that proposes that human well-being can best be advanced by liberating individual entrepreneurial freedoms and skills within the institutional framework characterized by strong private property rights, free markets, and free trade. (p. 2, italics added)

During COVID-19, many of the norms and techniques of marketization were questioned, but the crisis has also been seen, or at least explored, as a possible beginning of the end of neoliberalism as we know it. Harvey (2020) suggested, at the onset of the pandemic, that there were signs that the least neoliberal countries in the world (China, South Korea, and Singapore) managed the pandemic much better compared with countries like Italy or the United States. Furthermore, Harvey (2020) pointed out that the neoliberal economies are consumer-driven, which is deeply problematic in times of lockdowns, as such economies are dependent on high levels of economic growth. This is also true when it comes to certain market-oriented elements in the public transport systems. The incentivized contracts, whose aim is to increase the demand for public transport and discipline the bus companies, collapsed during the crisis as the market no longer grew, which we will analyze in more detail below. While analyzing the events during the spring of 2020, Harvey (2020) argued that the pandemic could be an event, a tipping point, in an uprising against the neoliberal system. He wrote:

Workforces in most parts of the world have long been socialized to behave as good neoliberal subjects (which means blaming themselves or God if anything goes wrong but never daring to suggest capitalism might be the problem). But even good neoliberal subjects can see that there is something wrong with the way this pandemic is being responded to’. (Harvey, 2020: 10)

Similar to Harvey’s (2020) analysis, Navarro (2020) claims that societies marked by neoliberalism, for example, with privatized healthcare systems, cuts in social expenses, marketization, and so on, have weaker capabilities compared with other societies when it comes to implementing measures to handle the pandemic. Standring and Davies (2020) as well as Nunes (2020) echoes this when arguing that the COVID-19 pandemic could be understood as a crisis of neoliberal healthcare systems, while also arguing that social mobilization and participation could radically transform these systems so they (again) operate for the public good. Drawing on a Schumpeterian long-wave perspective, Kılıç makes a critical analysis by pointing to the significance of turning points, which change the direction of investments in the economy. The COVID-19 pandemic could be seen as one such turning point and he substantiates this by stating that neoliberalism was already weak when COVID-19 hit in 2020. But what really could trigger the end of neoliberalism, is, Kılıç (2020) argues, whether COVID-19 creates a deep economic crisis that weakens its ideological foundation.

Yet, these critical analyses have been questioned. Although the COVID-19 pandemic had an effect on people’s daily routines and consumption patterns, such changes have not affected the ideology and institutionalization of neoliberalism, argue Briggs et al. (2020). In fact, Briggs et al. (2020) suggest that the wishes of governments and the big corporations of going back to ‘normal’, is actually a call for going back to neoliberal policies and institutions. Rather than addressing the root causes of the crisis and seeing this as a window of opportunity to change the system, governments merely treat some of the symptoms (Büscher et al., 2021). Since only the symptoms of this crisis are treated, Standring and Davies (2020) see little chances of transitioning toward a new path, beyond neoliberalism. Furthermore, Šumonja (2020) recognizes voices from liberal, right-wing, and neoliberal commentators, saying that ‘the state is back’ and that the market is playing a less important role in the economy and will do so in the foreseeable future.

But neoliberalism has always been built on an unruly coalition of free-market ideology and strong-state governance. COVID-19 has not changed this, both Šumonja (2020) and Briggs et al. (2020) argue. As in the financial crisis before, governments tend to bail-out big companies (to save the pillars of system) and pump tremendous amounts of money into the markets (so-called quantitative easing) in order to keep neoliberalism alive and preserve the free-market economies. According to Šumonja (2020), the COVID-19 crisis is a neoliberal crisis, but it will not result in the abolishment of neoliberalism. Most of the initiatives taken by governments are aimed at preserving the neoliberal regime of accumulation, instead of changing it radically. All this begs the question of whether this is also true for Sweden, and its marketized public transport sector. Has COVID-19 emerged as a tipping point in the marketized public transport system in Stockholm, or have neoliberal policies survived, once again, by incorporating and neutralizing critique?

## Marketized public transport: from regional monopolies to multinational corporations

In order to understand how the public transport market in Stockholm has grappled with COVID-19, we will first discuss how marketization works and how it is connected to a neoliberal regime of accumulation. When Birch and Siemiatycki (2016) define marketization, they say that ‘it involves the introduction of markets and market forces into the state, primarily into its functioning, authority and legitimation’ (p. 179). However, they do not say how this is done, or how authority and legitimation are translated into particular markets. Gane (2012), by contrast, elaborates a theory of neoliberal dominance and power that relies on the Foucault’s ideas on power exercised through discipline and knowledge, and how that can be translated into the neoliberal economic system. Gane (2012) argues that neoliberalism is upheld by governments through a normalization process of marketization. We will discuss this further below, when we discuss the *norms* and *techniques* propelling marketization (Araujo, 2007), but it is equally critical to understand that these *norms* and *techniques* are connected to the *representations* of markets (Kjellberg and Helgesson, 2007). Figures over the number of transactions, number of sellers, of goods sold, and so on are often used in *representations* of the market. Controversies occasionally emerge among the market actors when it comes to how the market is represented, not least if figures point to what might be perceived as inefficiencies or failures, which then cause debates about the *norms* and *techniques* governing the market (Araujo, 2007; Kjellberg and Helgesson, 2007).

While marketization is generally discussed in relation to larger infrastructure projects, the interest in public welfare services has been largely absent (see, for example, Birch and Siemiatycki, 2016; Hansen and Lindholst, 2016). For example, Farmer (2011) highlights how public transport has become ‘an entrepreneurial tool’ for neoliberal urban planning in Chicago. She shows that when public transportation investments are geared toward attracting tourists or affluent residents, everyday commuters are left with under-maintained infrastructures, whereby uneven urban development is intensified. Like many other scholars (see discussion in Birch and Siemiatycki, 2016), Farmer points to the neoliberalization of public transport *infrastructure* investments, without discussing the marketization of public transport *service* provisions as such.

Marketization is a term denoting the transformation of social functions into a market, but it does not necessarily mean the privatization of the responsibility for the social function, from a public to a private actor. Yet, marketization must be seen as part and parcel of a neoliberal regime of accumulation, although, as Mercille and Murphy (2019) claim, neoliberalism must first and foremost be seen as an ideological process translated into institutions. In so far as marketization is seen as an ideology or an established *norm*, an array of *techniques* is developed to promote competition, which is considered to tease out the ‘right’ incentives in neoliberal subjects (Madra and Adaman, 2014).

## Public procurement

In Stockholm, the bus traffic is procured on tendered contracts running 10 years or more. Öjehag-Pettersson and Granberg (2019) suggest that the spread and intensification of public procurement is a part of ongoing marketization processes. Although public procurement is central to understanding the practice of marketization, the relationships between procurement and marketization are surprisingly little studied (Öjehag-Pettersson and Granberg, 2019: 56; see also Brunsson and Jutterström, 2018; Hansen and Lindholst, 2016).

While public procurement is a *technique* of marketization in the public sector, the connections between them are gravitating toward competition. Marketization generally involves a process in which uniform systems are broken down into smaller bounded components, or geographical areas, in order to instill competition. Birch and Siemiatycki (2016) argue, for example, that

Marketization processes often involve the ‘unbundling’ of socio-technical networks or systems, which can be seen as monopolies (e.g. transport system), in order to open up different parts of the network or system to competition and competitive forces. (pp. 194–195)

When the system is unbundled and thereby broken down into smaller components, techniques are designed to promote competition (Hansen and Lindholst, 2016). According to Brunsson and Jutterström (2018), public procurement is one dominating *technique* enabling an organized form of competition. But pro-market policymakers are continuously searching for policy innovations that open up service provision to new actors, as well as new forms of cooperation and competition. Legislative reforms, with the goal of facilitating easy market-entry and increased competition, are often a prerequisite for unbundling service provision and instilling competition though public procurement.

## Incentivized contracts

Marketization in public transport has taken place via two waves of policy innovations. The first wave came when public transport authorities in Sweden decided to let private companies run the traffic on tendered contracts. This occurred during the early 1990s. The second wave came when the so-called incentivized contracts were introduced. Beginning in the 2000s, Stockholm and other regions moved from using contracts with fixed payments to bus companies (for ‘produced traffic’) to using contracts with incentives. Bus companies were then paid based on the numbers of passengers boarding their buses. These two waves of policy reforms aimed to transform public transport into a market and make the bus companies operate like businesses, with customers that needed to be satisfied.

While public procurement is a *technique* of marketization, incentivized contracts could be understood as a *tool* (Lascoumes and Le Gales, 2007). The introduction of this tool came with promises of greater flexibility for the bus companies, not least in planning routes and timetables. However, for the PTA, it has become difficult to change the transport system when the system is divided into different smaller geographical areas, or functional components, in which different companies compete with each other (e.g. Birch and Siemiatycki, 2016: 195).

Madra and Adaman (2014) suggest that economic incentives is *the* tool mobilized in neoliberal reforms. Drawing upon a historical analysis of the development of neoliberalism, they propose that neoliberalism is not so much about marketization as it is about incentivization (see also Mercille and Murphy, 2019). Of course, they are often coupled in practice. Because what markets are doing is providing the ‘right’ incentives by instilling competition. But incentivization may be decoupled from competitive markets, not least if public policy is based on systems of punishment. Yet, this idea of incentivizing all aspects of society, not least the public sector, makes markets and marketization initiatives appealing to many of the architects of neoliberal policies.

Competition triggers incentives, as one of the primary outcomes of competition is the separation of ‘winners’ from ‘losers’. As this incentivization is presented to be in line with ‘human nature’, going against marketization means, allegedly, going against ‘human nature’. Fisher has discussed the equation between neoliberalism, human nature, and the organization of social relations on the basis of self-interested actors, using the concept of ‘business ontology’. While Fisher (2009) suggests that this is how neoliberalism has established itself as ‘capitalist realism’, Madra and Adaman (2014) argue that neoliberalism amounts to a ‘depoliticisation through economisation’ (p. 692). Any attempt to ‘rethink the very organisation of economic practices that may be needed to break from the growth and austerity cycles of capitalism’ is silenced by referencing how such attempts jeopardize or go against ‘human nature’ (Madra and Adaman, 2014: 692).

This ideology of incentivization creates an economization of societies, of the public sector, and of social relations, making the social less valued than the economical and thus, decreases ‘human nature’ to the so-called homo economicus (Peetz, 2019). The governmentality of neoliberalism therefore understands only the language of economics, which implies that all individuals are equipped with the knowledge needed to take, economically speaking, the best decision based on the economic incentives at hand. Should opportunism emerge, which it always does in markets, regulatory interventions do not target ‘the opportunist economic agent *but* the mechanism designer—who possesses the expert knowledge to correct market failures by designing incentive-compatible institutions’. (Madra and Adaman, 2014: 701, italics added)

Summing up, we will now turn to public transport in Stockholm and explore how, and to what extent, the marketization of this sector came under scrutiny as part of the pandemic and whether the observations—or speculations—that we discussed above constitute one minor part of a larger turning point in the long wave of neoliberal accumulation. After all, due to COVID-19, the question of how the public transport markets are operating has been the subject of discussion: should it be organized based on competition through public procurement processes, and what techniques, including contract designs, should be used under these new conditions? These are the questions we will explore below.

## The public transport market in Stockholm during COVID-19

Up until the late 1980s, *Storstockholms lokaltrafik* (SL), the predecessor to the PTA, organized and operated all traffic in-house. SL moved toward relying on market mechanisms during the 1990s and in 2007 all traffic was procured. In 2006, the organization of SL changed. Previously, politicians were often deeply involved in micro-managing traffic operations (SL, 2002, p. 31; quoted in Bergström and Kahn, 2007). With the reorganization, the boundaries between the political level and the scope of influence of SL’s board as well as its top-management team, were specified. The politically appointed Board of Directors limited its scope of influence to the following: only to decide on matters of strategic importance and on large investments and procurement contracts (of EUR 25 million or more) (Bergström and Kahn, 2007: 10). The CEO and the top-management team reported to the Board of Directors, whose members were appointed by the owner: the politically governed Stockholm County Council (Bergström and Kahn, 2007).

In 2012, a new law governing public transport entered legal force in Sweden (SFS, 2010: 1065). The aim with the new law was to broaden the supply of operators in the market and strengthen competition through deregulation, which would increase the ridership. As part of this new law, regional Public Transport Authorities were formed in each of the 21 regions and counties, including Stockholm. The regional Public Transport Authorities were assigned a central role as they had to conduct market analysis, exercise market surveillance, and decide on the Public Service Obligations (PSOs), a concept deriving from EU directives. One of their first tasks was to produce a Regional Transport Supply Program, containing the Public Service Obligations. Again, this legislation remodeled the relationships between the public transport administration and the bus companies, as public transport was regarded as a market failure. Only where no commercial traffic could be foreseen, should PSOs be established by the administration in Stockholm. Based on the PSOs, the administration has divided the region into different geographical areas, and the bus companies compete with each other when bidding on the contracts in each of the areas.

The bus companies are multinational corporations. Many of them are owned by former state-owned railway companies. Keolis is jointly owned by SNCF (70%) and the Caisse de dépôt et placement du Québec (30%), Transdev, formerly Veolia, is owned by Caisse des Dépôts et Consignations (66%) and Rethmann (34%). Of the bus companies operating in Stockholm, only one is listed on the stock exchange: Nobina.

## ‘Do not travel by public transport’

When the Swedish Public Health Agency (*Folkhälsomyndigheten*) encouraged the population not to travel by public transport, passengers listened, and revenue dropped. Using its marketing channels, the regional PTA in Stockholm informed its travelers: ‘If you do not have to, do not travel by public transport’ (Heick et al., 2020). When interviewing Cederberg and Dahlént, both senior managers working at one of the major bus companies about the implications of this, they said that their entire ‘business model’, that is, to increase the modal share of public transport, had been ‘shaken up’ in its very foundation.

On 23 March 2020, the Nobina share halved its value. A month earlier, the share had been traded for SEK 78, but now it was traded at SEK 42. This mirrored the larger stock crash which occurred as part of the COVID-19 lockdowns. Yet, stock market analysts expressed confidence in the company and the market. Ericsson, one analyst at Avanza, said that:

The Nobina share has fallen sharply in the stock market crash of recent weeks. Bus traffic is in principle a completely cyclically independent activity, so the reaction may seem strange. [. . . ] The reason is that Nobina has a relatively large proportion of contracts with variable remuneration, where the number of passengers is an important parameter. Of course, these contain slightly more risk, but thus also better profitability and greater opportunities for revenue growth.

Nobina can use its expertise in bus traffic to optimise timetables and staffing. Qualitative parameters such as customer satisfaction and punctuality are also usually included in the remuneration models. Nobina has actively worked to increase the proportion of incentive-based contracts and has come the furthest in the Swedish market. Just under a third of the contracts in Sweden are incentive-based.

The proportion of compensation that is variable varies. Some contracts are fully variable while others are around 25-30 percent variable. [. . . ] In addition to the actual passenger numbers, Nobina of course has a lot of its own employed drivers who are at risk of becoming ill. Increased sick leave could lead to disruption and increased costs. (Ericson, 2020)

This analyst has insights both into how the public transport market operates and how Nobina is engaging and competing in that market. While it would be far-fetched to claim that the public transport market is embedded and governed by the logic of financialization (Christophers, 2015), obviously, this listed bus company was scrutinized as for its potential revenue streams, stemming from the incentive-contracts it is bound to.

As mentioned by the stock market analyst above, the contracts between the regional PTA in Stockholm (SL) and the bus companies are designed based on two metrics. First, the remuneration to the bus companies is partly based on how many people travel. Second, the remuneration is based on a combination of the number of vehicles used or the number of kilometers driven, and the number of hours each bus is circulating in traffic. Thus, when the number of passengers decreased, the revenues dropped for both the bus companies and the PTA. As the PTA maintained the number of departures more or less as before the pandemic, there was a widening gap in funding (Trafikförvaltningen, 2020b: 3).

## Not a decline in the rate of profit, but in revenue

During the 2008 crisis, governments bailed-out banks that were on the verge of bankruptcy to avoid a larger collapse in the financial system. A similar pattern emerged in 2020 in Stockholm, but on a smaller scale. Due to the restrictions on mobility, bus companies’ revenue-margins were ‘all blown away’, Adolfsson, the CEO of one of the largest bus companies, told us. In order to save the bus companies and ultimately the market, strategies quickly developed to mitigate the financial impact of COVID-19. Two strategies took form. The first focused on getting people on board the buses again in a safe and secure way. Crowding on buses and various protective measures were discussed among the PTAs and the industry-based *National Bus Federation*. The second strategy focused on ensuring short-term profits. While revenue dropped, the bus companies and the conservative leadership in Stockholm worked together to make the national government guarantee subsidies, which would make up for the losses in revenues and ensure profits.

In March and April 2020, dialogues began between *National Bus Federation, Sweden’s Municipalities and Regions* (SKR) and *Swedish Public Transportation* (the association of public transport authorities in Sweden). One key question in those dialogues centered around how increased crowding on buses would be handled now that the transport supply had been cut. Another key question concerned subsidies to ensure profits. Public and private interests joined forces when the public authorities in Swedish Public Transportation and the private bus companies in the National Bus Federation both demanded financial support from the national government for maintaining public transport operations. Tamsons, the conservative politician from the ruling party Moderaterna in the Region of Stockholm, did not hold back on the historical significance of the crisis, when he on 20 May 2020 said the following:

Ronald Reagan once said that the most frightening words that exist are ‘I am from the state and I am here to help you’. However, public transport needs to hear those words again. The government’s announced support for public transport was welcome but is far from sufficient. A guarantee is needed from the state for full compensation for the social efforts that are now being carried out. (Moderaterna, 2020)

Around SEK 1 billion a month was lost in ticket revenues; Swedish Public Transport estimated the cost of operating traffic as usual, but without all the usual passenger volumes. It would be about 8–10 billion kronor in deficit during the whole year. As shown in the quote above, Moderaterna demanded increased support from the national government. Politicians in the government, not least the members of the Riksdag’s (Sweden’s national parliament) Transport Committee, also wanted to provide state support to public transport.

Due to the expected losses for all the major bus companies, the National Bus Federation contacted the Swedish Public Transport and asked them to prepare a common solution for all the regions in Sweden. Swedish Public Transportation was reluctant to take such a responsibility. The reason, we were told by Franzen, the vice-director of the National Bus Federation, was that each region handled and designed these types of contracts in their own ways. Ebeling, the CEO of one of the major bus companies echoed this. This was also a sensitive issue for the bus companies, we were told, as the bus companies are not allowed to cooperate with regard to agreements and contracts as that could hamper competition in the market.

Spring turned into summer. Bilateral discussions between the bus companies and the PTA in Stockholm and in the other regions continued. SL, the PTA in Stockholm, announced in August 2020 that the reduction in travel demand continued. From having decreased by 20% in the spring and 35% during the summer, travel in August had fallen to 55%–60% compared with the corresponding period in previous years. In order to be able to financially compensate the bus companies, SL signed new interim supplementary agreements during the month of April for the period 1 April to 30 September ‘to handle compensation and traffic ordering procedures’ (Trafikförvaltningen, 2020b: 5). In practice, the use of incentivized contracts was temporarily replaced by a production-based remuneration model. This meant that the PTA took over all financial risks.

### Shared view, different solutions

Although the bus companies had a shared view of the consequences of the pandemic—that revenues dropped as passenger volumes decreased—they dealt with the consequences in slightly different ways and drew partly different conclusions. Cederberg and Davidsson, both senior managers, told us that they see themselves as excelling in planning and operating traffic, but not in managing the PTAs overall risks. This comment was an implicit critique of how some PTAs in other parts of Sweden understood the incentivized contracts. The director of Skånetrafiken wrote in a hotly debated piece on LinkedIn that the bus companies wanted to benefit from the upsides of incentivized contracts, but not the downsides. Adolfsson, the CEO of one of the larger bus companies, opposed this, and told us that the bus companies should not ‘act as insurance companies for the public transport administrations’.

We were also told by Beckman, another CEO, and again by Cederberg and Davidsson, that attempts to renegotiate how the remuneration models would be applied, were not that successful. However, several of the CEOs emphasized that the PTA in Stockholm, SL, handled the crisis with a ‘business-like’ mindset as SL wholeheartedly wanted to financially support the bus companies.

Ewerstrand (2020), another stock market analyst following Nobina, commented in November 2020 on the company’s results for the broken financial year 2020/2021, that the pandemic could affect the company in the long run:

In a very negative scenario, Nobina could lose most of this incentive-based income if more people choose to work from home even after the pandemic. This is also a revenue risk that has been discussed for the commercial real estate companies with a significant proportion of office properties. An aspect to consider is that those who use the bus for commuting to work are to a large extent healthcare and care-home staff, school staff, manufacturing and shop employees who may not have parking facilities or think that congestion charges are too high in metropolitan areas. The above occupational categories, which work with people, cannot work from home to any great extent. In addition, young new employees, students, immigrants (who have not had time to obtain a Swedish driving license), and pensioners are other important groups who regularly use the bus. [. . . ] Finally, if we are to sum up Nobina, there is a certain risk with incentive-based income, but it should also not be exaggerated as many social groups that use public transport cannot work from home.

Despite the length of this quote, it is worth keeping in its entirety because it shows how cash is understood to flow in the public transport market. Since the majority who travel by public transport can neither take the car to work, nor work from home, the loss of revenue for the bus companies would not be substantial, Ewerstrand summed up his ‘buy’ recommendation. Although there are obvious risks with incentive-based contracts, this technique is obviously appreciated by this particular stock market analyst as it is disciplining the bus company, in this case Nobina. For the past decade, many were positive towards incentivized contract designs. Ingemarsson, one civil servant we interviewed, said that:

‘We were very positive [to incentive-based contracts] and I think the industry was too, precisely for the reason that it drives quality development, because you want to be involved and make more money if you have the opportunity’. (Ingemarsson)

Looking ahead, two of the CEOs told us that they wanted to see production-based contracts, that is, contracts in which remuneration is based on the number of buses and hours the buses circulate in traffic. This is how contracts used to be designed earlier, in the 1990s and early 2000s. However, these CEOs also told us that they did not want to ‘go back’ to how these production-based contracts looked 20 years ago. Instead, they wanted to see new ‘updated production-based’ contract designs, where quality parameters are rewarded to a greater extent. One thing the pandemic has showed is that incentive-based contract designs are too uncertain, we were told. While discussing risks, Beckman explained that the risks with a pandemic are difficult to calculate and therefore also to put a price on. Incentive-based contract designs often have a base-year, from which increases in traveling are benchmarked. But due to the pandemic, it is unclear which year should be used as the base-year, Beckman told us. If the PTAs choose a base-year with a relatively low number of passengers, then the PTAs are afraid that the bus companies will have a huge upside when commuters return to ‘normal’ levels, Adolfsson and Beckman, both CEOs but for different companies, told us separately.

Previously, before the pandemic, the bus companies purchased buses with an overcapacity at the beginning of a contract (often valid for a 10-year period) to take account of an increase in ridership toward the end of the contract. It is doubtful whether this will happen in the future, Ebeling stated. Nobody knows how travel is affected by the pandemic in the long run. Often, bus companies make a financial deficit in the first years of the contracting period, but only to make a profit toward the end. The price put in the tender is an average of this. Now, we were told by all of the CEOs, this way of calculating will not work in the future.

## Ensuring a future of marketization

Pricing of risks is obviously key when placing bids in the procurement process. Many of the CEOs told us that COVID-19 has turned pricing strategies upside down. If the bus companies must start pricing risks such as global pandemics, then the pricing would be 15%–20% higher than today, all other things being equal. This was repeated by the bus companies’ CEOs and senior managers, including Adolfsson, Cederberg, and Davidsson. These CEOs were also outspoken when it came to the regional politicians’ level of knowledge of how the public transport market operates. To remedy this lack of knowledge, representatives from the bus companies have been ‘training’, a word used by Beckman, both regional politicians and senior officials of how the market operates. ‘They [the politicians] must understand’, said Cederberg, ‘that the bus companies deliver a community service that they have priced according to the expected increase in population and travel demand’. Starting to internalize the costs of a global pandemic would cause prices to skyrocket, she added. Stockholm is the exception here, though, as SL had a ‘business-like’ mindset and the responsible politician, Tamsons, called for state-intervention early on.

We were surprised to hear those incentivized contracts were *not* designed to provide the bus companies with a bonus if performing well. ‘Incentives are not a bonus that is *in addition* to ordinary compensation’, argued both Cederberg and Davidsson. Another CEO, Beckman, similarly said that regional politicians seem to believe that incentives are there *in addition* to a basic allowance, but this is not the case. Due to the incentives, the bus companies have lowered the prices in their bids as a competition strategy. Competing by offering the lowest bid-price and then hoping for enumeration based on incentives to cover up the lower bid-price, has become commonplace among the companies. A third CEO, Ebeling, said that approximately 30%–40% of the company’s revenue was linked to incentives and therefore it was important to solve the problems with the reduction in travel and revenue in dialogue with the public transport authorities. In practice, however, there was no dialogue. SL, as the other public transport authorities in Sweden, simply told the bus companies how they wanted the contracts to look and be applied—and that is what then was decided.

At the state level, the Transport Committee in Riksdagen, the national parliament in Sweden, discussed how to support public transport. SKR and the National Bus Federation calculated losses of the order of SEK7.5 billion in 2020. The government support eventually landed at 5 billion (3 billion allocated in 2020 and 2 billion allocated for 2021). However, the loss in 2021 was estimated to land at 5–8 billion. In Sweden, the support came relatively late, while the Danish and Norwegian bus companies received state-aid early on. Such differences in the timing of state-aid in the Nordic public transport markets could distort competition in the long run. Franzen, working for the National Bus Federation, said this could lead to a situation where small- and medium-sized bus companies in Sweden do not survive the pandemic, whereas Danish and Norwegian competitors may more easily enter the Swedish market.

Gidlund, one of the politicians in the Riksdag’s Transport Committee, stated that it is not easy for the government to support the public transport sector because this is a regional issue, and regions have a large degree of freedom in Sweden due to the principle of self-government. Also, the Riksdag cannot influence how contracts between the public transport authorities and the bus operators are designed. Having said that, Gidlund explained that the national government, of course, supports public transport through the state budget and through various financial support mechanisms for public transport infrastructure.

## Discussion

This study set out to explore how COVID-19 has impacted the public transport market in Stockholm. We did so by studying how the bus companies that operate the traffic on behalf of SL grappled with the lower levels of ridership and loss of revenue. When exploring these issues, we have been making connections with discussions on marketization as a *norm* and public procurement and incentivization as *techniques* (e.g. Madra and Adaman, 2014). Both the *norm* and *techniques* came under pressure during the pandemic, but we also wanted to explore whether this could be understood as the beginning of the end of neoliberalism, or if neoliberalism somehow have managed to reinvent itself and come out afresh again after the crisis (Harvey, 2020; Kılıç, 2020; Navarro, 2020). By zooming-in on one particular sector that has gone through two waves of marketization reforms, it would be possible, we thought, to assess whether cracks in the *norms* and *techniques* of marketization could be seen as cracks in institutionalized forms of neoliberalism, that is, in marketized public transport service provision in Stockholm.

From this study, the following patterns emerge. To begin with, how the public transport market is performing is made visible through *representations* of the market (Araujo, 2007; Kjellberg and Helgesson, 2007). This includes, for example, statistics on the number of travelers, the number of boarding passengers, and associated costs. Such representations recurred on several occasions during the turbulent year 2020, both for ridership and ticket revenue, although the data were initially many times rough estimates. Crossed-checked figures came later in the year. Although the numbers of travelers declined dramatically, the bus companies fared quite well as the financial results turned out to be not as negative as had been estimated during the spring of 2020.

There are relatively established *norms* for how the public transport market should function. While the public transport authority acts as a buyer and the bus companies as a seller of an unbundled service (Birch and Siemiatycki, 2016), competition is guaranteed through the bidding in the public procurement processes (Öjehag-Pettersson and Granberg, 2019). But did the pandemic lead to a questioning of these norms? And what strategies did the bus companies utilize to ensure profits, even in a situation with lower levels of revenues? While glimpses of collaborative attempts could be seen between the bus companies on one hand and the regional public transport authorities on the other, not least when it came to obtaining subsidies for maintaining traffic operations, competition was still the dominating *norm*. Collaboration in fact centered on ensuring the future of marketization. This is in line with how self-regulating markets are supposed to operate under neoliberalism, where markets underwrite competition, which in turn is disciplining market actors by creating ‘the right’ economic incentives (Madra and Adaman, 2014; Mercille and Murphy, 2019). In the public transport market in Stockholm, the *norms* are translated into *techniques*, most notably public procurement and incentivized contract design. These techniques regulate the exchanges and also the responsibility of each party involved. But those techniques also form and institutionalize neoliberalism in so far as public transport is now understood and treated as if it was a market, with buyers and sellers of what is supposed to be clearly demarcated services (see Madra and Adaman, 2014; Mercille and Murphy, 2019).

While the market norms so far have remained unquestioned, COVID-19 led to a re-evaluation of one of the techniques: incentivized contracts. Due to the different contract designs in each region, a common solution for all regions was unfeasible. Although the market *norms* were the same across the regions, the *techniques* differed and therefore also ‘the solutions’ on how to renegotiate the incentivized contracts. The regional Public Transport Authority in Stockholm showed great understanding of the bus companies’ business models, according to the CEOs we talked to, as the region promised to cover all costs and pushed for state-aid. Running ordinary traffic with far less passengers would be costly, but this would be paid by the region or through state-aid, leading politician Tamsons claimed.

What strategies did the bus companies then pursue to protect the institutionalized forms of marketization discussed here? The bus companies worked on informing or ‘training’ politicians and officials on how the incentivized contracts work and what these contracts aim to achieve (i.e. increased traveling). We take this to mean that this particular *technique* could be improved, but that more knowledge—or rather better *representations* of how the market performs—is needed. The reason the technique of incentivization was criticized by two of the bus companies was fairly straightforward: they were too risky and putting a price on those risks would not make them competitive in future bidding processes. Compared with other welfare markets (see, for example, Brunsson and Jutterström, 2018), public transport markets are shaped by its dual income streams: public subsidies coming from taxes on one hand, and ticket revenue coming from travelers on the other hand. When ticket revenues dropped during COVID-19, tax subsidies had to go up, otherwise operations would not be able to continue.

What we have shown is how marketization was saved from itself through two interconnected strategies: first by joining forces across the public–private divide and collaboratively asking for state-aid and, second by renegotiating contracts bilaterally between the public transport authorities and bus companies to ensure future profitability. As this shows, the *norms* surrounding the marketization of public transport were not criticized, but rather the *technique* of incentivization.

Returning to our earlier discussion, neoliberalism has been very much kept alive even during COVID-19, as both Šumonja (2020) and Kılıç (2020) have argued. Although it is not yet clear how permanent the travel recommendations will be as of summer 2021, or how significant the consequences for travel will be in the long run, the pandemic has not substantially affected the *norms* for organizing the public transport market in Stockholm. Instead of questioning the *norms* for how the market should operate, developing and improving the *techniques* emerged as a crucial strategy for the bus companies. This contributes to Šumonja (2020) argument that the COVID-19 pandemic does not bring neoliberalism to an end, as prophetically suggested by Harvey (2020), but rather, shows how the adjustments were made to save marketization from collapsing. Although it might be a bit too premature to draw long-term conclusions, our analysis of this particular public transport market suggests that the pandemic did not constitute a critical event or a ‘turning point’ in the long wave of neoliberal accumulation (cf. Kılıç, 2020).

While many scholars have pointed to neoliberalism’s capacity to absorb and neutralize critiques, Slobodian and Plehwe (2020) point out in the introduction to the edited volume, *Nine Lives of Neoliberalism*, that there are essentially ‘two ways of making sense of neoliberalism’s longevity’ (p. 2). While one refers to ‘the durability of the blocs of capital and their allies in government’, the other refers to ‘the expansion and adaptation of neoliberal worldviews encroaching upon the competing ideologies of conservatism and social democratic liberalism’. What we have done here is to show how neoliberalism operates, how it is saving itself despite itself, all in the midst of a global crisis. By protecting established marketization norms, by re-evaluating the public procurement techniques, as well as by abandoning one key technique (incentivization) used earlier to boost profitability, the market actors managed to save the market from collapsing. Our detailed empirical exploration has contributed with new knowledge on neoliberalism’s longevity by showing which strategies the bus companies used, and to what extent the public transport authority in Stockholm supported these.

Consequently, even though neoliberal policies have come under enormous distress due to the COVID-19 pandemic, the crisis has been more of an exception than a ‘turning point’ in the public transport market in Stockholm. We are aware that the notion of ‘turning point’ is a metaphor and the implications of COVID-19 might not materialize until much later. Yet, developments during the spring and summer of 2021 have substantiated our analysis. This also points to the observation that a global pandemic is not enough to halt neoliberalism. It is global political activism at the local scale, not a worldwide socio-biological crisis, that must be mobilized to achieve political change.

## Conclusion

The COVID-19 pandemic has made it more difficult to use certain neoliberal tools for organizing public transport, such as incentivized contracts, while others, like public procurement, remain unquestioned. One might even say incentivized contracts has reached a dead-end policy-wise, which could mean that this form of marketization is on its way out. But does this also imply that we start to see some cracks in the hegemony of neoliberalism? This is something Harvey (2020) has discussed and Kılıç (2020) echoed this when suggesting that the pandemic might constitute a ‘tipping point’ in the neoliberal order. However, neoliberalism, just as Šumonja (2020) argued, has never been about pushing out the state, but rather using it to create ‘more’ and ‘better’ markets.

The bus companies that operate public transport in Stockholm are now trying to change the incentivized contracts so that they still can make a profit by providing this public service. As our case study has shown, the techniques of marketization are questioned, but the norms governing the market, that of competition, remain in firm place. Neoliberalism, as we see it, is still holding on to its grip on public transport in Stockholm, and it is similar in other cities in Europe. After all, neoliberalism has shown to be rather resilient to crisis, as Harvey (2020) pointed out. Because neoliberalism is built around the idea of incentivization, and markets organize social relations along the lines of competition, neoliberalism is quite elastic, yet deeply embedded in social institutions.

However, as mentioned previously, certain *techniques* of marketization are not as useful during and maybe also after the COVID-19 pandemic. This could be a sign that neoliberalism’s influence on public transport in terms of marketization is decreasing. But we also see that the *norms* of marketization are largely unquestioned, whereby neoliberalism will most likely also survive this crisis. One strategy used by the bus companies is to ‘educate’ politicians and officials in order to change the market *techniques* in favor of their situation. The imminent question for the public transport administrations in Sweden, but certainly also in other countries, will be how national or subnational governments will react. If national governments intervene, this might be part of a ‘tipping point’, though social transformations will not happen without grassroot mobilization in social movements. National or regional governments may seize the moment and change the system, if not completely, then at least to some degree so that the marketization of public transport diminishes. Rather than safeguarding the profitability of the bus companies, as in the case in Stockholm, other values, such as equity, justice, sustainable mobility, accessibility, and democracy should be at the center (e.g. Peetz, 2019).

## Appendix 1

### List of interviewees

Adolfsson: CEO at one of the larger bus companies

Beckman: CEO at one of the larger bus companies

Cederberg: Market director at one of the larger bus companies

Davidsson Director of business development at one of the larger bus companies

Ebeling: CEO at one of the larger bus companies

Franzén: Director at the National Bus Federation

Gidlund: Member of parliament, Traffic Committee

Hansson: Public Transport, Civil servant,

Ingemarsson: Public Transport, Civil servant

Juhlin: Public Transport, Civil servant

## Notes

1. We are fully aware that neoliberalism is a contested concept and that there is rich discussion on the viability of this concept as an analytical category, see, for example, Simon Springer et al. (2016) and Mirowski and Plehwe (2009). As many of these scholars point out, the debates on the concept’s meaning should not disqualify its analytical value, at least if used in a way that does not lump all ‘bad’ things (one does not like) into this conceptual category.
